# Surgical treatment of intra-articular calcaneal fractures: description of a technique using an adjustable uniplanar external fixator

**DOI:** 10.1007/s11751-014-0207-x

**Published:** 2014-12-25

**Authors:** Randal Rudge Ramos, Carlos Daniel Candido de Castro Filho, Roger Rudge Ramos, Cíntia Kelly Bittar, Mario Sérgio Paulilo de Cillo, Carlos Augusto de Mattos, José Luís Amim Zabeu, Antenor Rafael Mazzuia

**Affiliations:** Celso Pierro General and Maternity Hospital, Pontifical Catholic University of Campinas, PUC-Campinas, Av. John Boyd Dunlop, s/n, Jardim Ipaussurama, Campinas, SP Brazil

**Keywords:** Calcaneus, Os calcis, External fixator, Intra-articular fractures

## Abstract

Several surgical techniques are available for the treatment of intra-articular calcaneal fractures. The use of a uniplanar external fixator is an option for the treatment of fractures classified as Sanders types 2 and 3. Satisfactory reduction and stabilisation of the fracture are achieved by means of mini-incisions and fixator adjustment. The advantages of this technique include less soft-tissue damage, avoidance of internal implants and early weight-bearing with the potential to improve postoperative recovery.

## Introduction

The treatment of displaced intra-articular calcaneal fractures is difficult and controversial [1]. Recent studies emphasise the accuracy of diagnosis, classification and precise indications for surgery [1–3]. Several treatment strategies are suggested for this type of fracture: conservative, including cast immobilisation and Harris’ traction; surgical, including percutaneous pinning, open reduction and internal fixation, ostectomy, primary arthrodesis and external fixation [2–5].

Complex hindfoot anatomy and the associated biomechanics pose a challenge to the surgical treatment of displaced intra-articular calcaneal fractures because the calcaneus is continuously subjected to compressive forces with the articular facets participating in both Chopart’s joint of the midfoot and in the subtalar joint with the talus [1–3, 7]. Fractures in this area may therefore lead to limitation in work and everyday activities [2].

The development of computed tomography has added to the assessment of these complex intra-articular fractures; together with use of modern orthopaedic implants, there have been improvements in the planning and performance of surgical procedures aiming at their stabilisation [2, 3, 7]. Although open reduction with internal fixation is considered the treatment of choice in Sanders fracture types 2, 3 and 4, there is no consensus on the final results of either surgery or conservative treatment [1, 4–6]. The aim of treatment of displaced intra-articular fractures is to restore the structure in three dimensions, with special emphasis on the proper alignment in the axial and coronal planes and on the height of the calcaneus [2, 4, 7–9]. Nevertheless, some degree of subtalar joint stiffness and degeneration always remains following an articular fracture, regardless of the treatment performed [6–9].

The use of mini-incisions and external fixation has been shown to be a reliable technique in achieving a stable reconstruction of calcaneal fractures [10]. The clinical results are comparable to the results of the open reduction and internal fixation technique [6–10]. External fixation has several advantages: It is less invasive, the time of surgery and hospitalisation is shorter, it allows for early weight-bearing on the affected limb, and it has a lower risk of complications as compared to the open technique [6–10].

## Materials and methods

This is a case series involving seven patients (all male and manual labourers) of whom three had bilateral fractures. There were ten intra-articular calcaneal fractures sustained from falls from heights from January 2010 to December 2012. No patient had significant comorbidities (concomitant other diseases or a smoking habit) that would undermine the proposed treatment. Surgery was undertaken within 2 weeks after the injury. A mini-incision was used for all cases. Stabilisation using a calcaneal uniplanar external fixator was used for Sanders types 2 and 3 calcaneal articular fractures. The contraindications for this technique are:

1. A long interval from trauma to surgery.
2. A poor state of the soft tissues at the mini-incision site.
3. Sanders type 1 and 4 fractures.

Outcome measures were through the AOFAS scale and radiographic parameters. These are tabulated (Table 1).

**Table 1:** Outcomes

Patients (age and occupation)	Bohler angle		Gissane angle		Calcaneal width		AOFAS
	Preop	Postop	Preop	Postop	Preop (cm)	Postop (cm)	
45 years painter	−20°	0°	190°	110°	5.6	5.4	70
52 years electrician	8°	30°	150°	105°	5.6	5.2	87
47 years bricklayer	8°	24°	128°	110°	5.2	4.4	84
35 years driver	0°	12°	194°	130°	4.8	4.0	77
53 years bricklayer	−20°	22°	180°	130°	6.2	5.5	86
Contralateral side	−5°	18°	90°	120°	5.9	5.7	67
61 years retired	0°	18°	100°	115°	5.0	4.8	77
Contralateral side	10°	32°	120°	100°	5.4	5.2	70
53 years builder	−10°	10°	94°	114°	4.8	4.5	63
Contralateral side	6°	12°	162°	126°	4.8	5.0	73

### Surgical technique

Imaging necessary for preoperative planning includes lateral and Harris axial heel radiographs and computed tomography. Additionally, an assessment of the soft tissues of the foot is important.

**Fig. 1:**
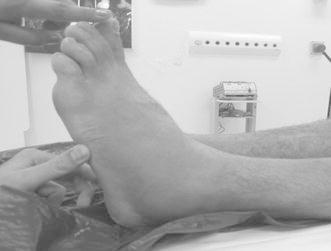
Patient in the supine position

The patient is placed in the supine position with a cushion below the ipsilateral gluteal region (Fig. 1). In case of bilateral calcaneal fractures, the patient is placed in the prone position. A fluoroscopy device is placed to acquire lateral and axial calcaneal images, and a tourniquet is placed and inflated in the proximal region of the limb.

**Fig. 2:**
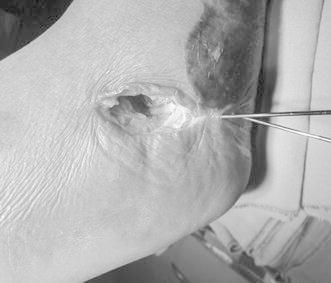
Lateral mini-incision

Approaching the sinus tarsi, a 3-cm mini-incision is performed. Haemostasis and dissection are performed to allow for better visualisation of the fracture (Fig. 2). A bone elevator is placed to rise and adjust the fragments that constitute the subtalar joint and form the Bohler’s angle. Following the reduction of the subtalar surface, the fragments are held temporarily using 1.5 Kirschner wires.

Application of the external fixator follows. The pin guides are inserted into the clamps and tightened to keep them parallel. The first Kirschner wire is placed through its wire guide positioned anterior to the subtalar joint and flush with the subchondral talar bone in the direction of the sustentaculum tali. A second wire is placed in the lateral joint fragment using the same procedure, and now both the anterior and posterior fragments are held. Following fluoroscopic confirmation of the position of these wires, the first pin is inserted through the empty guide in the clamp either using a drill or manually. At this stage of pin placement in the calcaneum, varus or valgus deformity may be corrected by inserting a pin at an angle through the clamp to act as a support. Again by using the pins as a joystick, it is possible to achieve proper alignment of the body of the calcaneus with the articular fragment fixed to the first clamp.

**Fig. 3:**
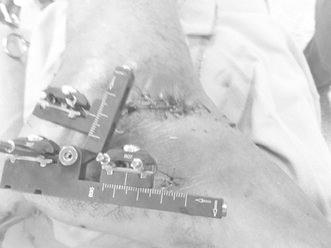
Positioning of the external fixator

The remaining pins are then inserted, and the pin guides are removed. With all pins in correct alignment, the external fixator is then locked onto the pins with particular attention paid to the distance between the device and the skin and keeping in mind the occurrence of postsurgical swelling (Fig. 3).

**Fig. 4:**
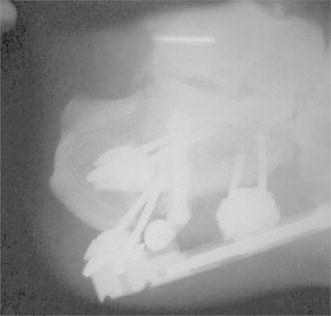
Postoperative radiograph on lateral view

**Fig. 5:**
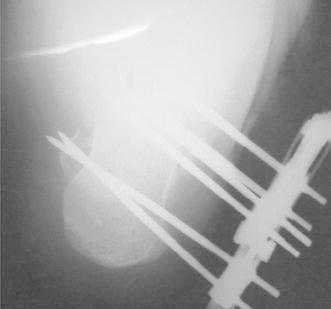
Postoperative radiograph on axial view

The main rail is distracted first, separating the anterior and posterior fragments and unlocking the comminuted fragments of the depressed area. The amount of distraction usually varies from 5 to 10 mm. Following the distraction of the main rail, distraction of the subtalar arm is performed until resistance is felt. The calcaneal lateral view should reveal a parallel middle and posterior facet, and the lateral view should show reductions of the Bohler’s and Gissane angles. The fixator’s position and the fracture reduction are observed by fluoroscopy (Figs. 4 and 5). There was no need for adjustments during the procedure.

### Postoperative management

Early mobilisation of the ankle and subtalar joint is indicated, i.e., during the first postoperative week. The sutures are removed 2 weeks after surgery. The patients begin weight-bearing 4 weeks after surgery. The fixator is removed in week eight, and physical therapy is then started.

## Results

Radiograph assessment postoperatively showed an average Bohler’s angle of 13.5°, with the angle of Gissane measuring was 118.3°; the normal range for Bohler’s angle is 20°–40° and 100° for the angle of Gissane [1].

On the AOFAS, score results are considered excellent if from 90 to 100 points; good if between 80 and 89; fair if between 70 and 79; and poor when <69. In all three bilateral cases, we had in one of the sides poor results in AOFAS scale due to more severe injury on these sides. Only one patient had infection in the wound area, involving only skin and subcutaneous tissue, which resolved promptly with antibiotic use, without the need for surgical debridement.

## Discussion

 Calcaneal articular fractures are severe injuries usually associated with permanent and disabling sequelae [2]. As they mostly affect young individuals, particularly men within the economically active age range, they can cause serious socioeconomic losses.

The use of a uniplanar external fixator associated with a mini-incision [3] allows for satisfactory results without performing internal synthesis, thus reducing discomfort, bulging of the synthesis material, and the need for a second surgical procedure to remove the implant.

As the soft-tissue injury is small, the risks of skin necrosis, suture dehiscence and infection are lower compared to the traditional surgical methods [3, 6]. Nevertheless, this technique demands broad experience and thorough anatomical knowledge on the part of the surgeon, as the small incision only allows for direct visualisation of the subtalar region; thus, fluoroscopy is often needed to achieve proper reduction of the fracture. Once properly installed, the calcaneal external fixator might later be adjusted to correct the varus deviation and to perform fragment distraction to achieve better reduction.

Patients treated using the calcaneal external fixator technique tend to recover very quickly. The fixation allows walking with partial weight-bearing starting in week four, and subtalar and tibiotalar joint mobility is stimulated during the immediate postoperative period.

To ensure the success of the procedure, in addition to the criteria inherent to any surgery involving the calcaneus, the surgical staff must be thoroughly acquainted with the specific materials and instruments that are used. Due to the small number of cases and the high cost of implants, other therapeutic options must be chosen when the above-mentioned criteria are not met.

Mini-incision and external fixation is a reliable technique to achieve stable reconstructions of calcaneal fractures. The clinical results are comparable to those obtained with the open reduction and internal fixation technique. As advantages, mini-incision and external fixation are a less invasive procedure, the time of surgery and of hospitalisation are shorter, and the risk of complications is lower compared to the open technique. Scientific papers were not found available in order to make comparisons.
